# A High-Quality, Long-Read *De Novo* Genome Assembly to Aid Conservation of Hawaiiʻs Last Remaining Crow Species

**DOI:** 10.3390/genes9080393

**Published:** 2018-08-01

**Authors:** Jolene T. Sutton, Martin Helmkampf, Cynthia C. Steiner, M. Renee Bellinger, Jonas Korlach, Richard Hall, Primo Baybayan, Jill Muehling, Jenny Gu, Sarah Kingan, Bryce M. Masuda, Oliver A. Ryder

**Affiliations:** 1 Department of Biology, University of Hawaii at Hilo, Hilo, HI 96720, USA; mhelmkam@hawaii.edu (M.H.); belling@hawaii.edu (M.R.B.); 2 Institute for Conservation Research, San Diego Zoo, Escondido, CA 92027, USA; CSteiner@sandiegozoo.org (C.C.S.); ORyder@sandiegozoo.org (O.A.R.); 3 Pacific Biosciences, Menlo Park, CA 94025, USA; jkorlach@pacificbiosciences.com (J.K.); rhall@pacificbiosciences.com (R.H.); pbaybayan@pacificbiosciences.com (P.B.); jmuehling@pacificbiosciences.com (J.M.); jgu@pacificbiosciences.com (J.G.); skingan@pacificbiosciences.com (S.K.); 4 Institute for Conservation Research, San Diego Zoo Global, Volcano, HI 96785, USA; bmasuda@sandiegozoo.org

**Keywords:** runs of homozygosity (ROH), inbreeding depression, major histocompatibility complex, toll-like receptors, behavior, SMRT sequencing

## Abstract

Genome-level data can provide researchers with unprecedented precision to examine the causes and genetic consequences of population declines, which can inform conservation management. Here, we present a high-quality, long-read, *de novo* genome assembly for one of the world’s most endangered bird species, the ʻAlalā (*Corvus hawaiiensis*; Hawaiian crow). As the only remaining native crow species in Hawaiʻi, the ʻAlalā survived solely in a captive-breeding program from 2002 until 2016, at which point a long-term reintroduction program was initiated. The high-quality genome assembly was generated to lay the foundation for both comparative genomics studies and the development of population-level genomic tools that will aid conservation and recovery efforts. We illustrate how the quality of this assembly places it amongst the very best avian genomes assembled to date, comparable to intensively studied model systems. We describe the genome architecture in terms of repetitive elements and runs of homozygosity, and we show that compared with more outbred species, the ʻAlalā genome is substantially more homozygous. We also provide annotations for a subset of immunity genes that are likely to be important in conservation management, and we discuss how this genome is currently being used as a roadmap for downstream conservation applications.

## 1. Introduction

Whole-genome sequencing of threatened and endangered taxa enable conservation geneticists to transition from a reliance on limited numbers of genetic markers toward increased resolution of genome-wide genetic variation [1,2]. Such genome-level data offer unprecedented precision to examine the causes and genetic consequences of population declines and to apply these results to conservation management (reviewed in [3,4]). Moreover, continued decreases in the costs of genomic sequencing technologies make this information increasingly available for non-model organisms, including those with large genomes (e.g., [5,6]; also see [7,8,9] for cost comparisons across platforms). Although challenges remain for bridging the gap between generating genomic data and applying this information to species management, this gap continues to close (for detailed discussions, see [3,10,11,12,13]).

Here, we describe long-read, whole-genome sequencing and *de novo* assembly for the critically endangered ʻAlalā. With 142 birds alive as of March 2018, this species is one of the most endangered avian species to have its genome assembled. The genome now provides valuable resources for conservation efforts, such as positional information and sequence data for candidate genes that are likely to have important fitness consequences (e.g., genes associated with immunity, mate choice, learning, and behavior). A high-quality genome also provides a tool for developing and mapping large numbers of genome-wide markers (e.g., single nucleotide polymorphisms; SNPs), which will improve estimates of relatedness and individual inbreeding coefficients (e.g., [14,15,16,17]). Improved relatedness estimates will be important for choosing mating pairs in the conservation-breeding (i.e., captive-breeding) program, where inbreeding depression (i.e., loss of fitness due to inbreeding) has been observed during pedigree analysis [18]. The genome will also be valuable for comparative studies aimed at understanding the evolution of tool use and other behaviors (e.g., [19]). Such comparative genome analyses could be especially important for conservation purposes, as they offer the potential to identify the genetic basis of fitness-related traits, both within and across species.

### Study Species and Aims

Historically widespread within mesic and dry forest habitats on the Island of Hawaiʻi, the ʻAlalā population declined rapidly during the twentieth century. Likely causes for the decline include habitat destruction and introductions of avian diseases and ungulates [20,21]. By 1970, the population was estimated at fewer than 100 individuals, at which point a small-scale conservation-breeding program was initiated. During the 1990s the total number of birds declined to less than 20, and in 1996 the last wild individual to supplement the newly modernized, more intensive breeding program was collected [18]. In 2002 the species became extinct-in-the-wild, but by then the number of birds in the breeding program was increasing (reviewed in [22]). Today, the ʻAlalā is one of the most endangered endemic bird species in Hawaiʻi, having existed entirely in captivity from 2002–2016 [18]. All extant individuals are descended from nine genetic founders that established the conservation-breeding program [21,22]. In 2016, a long-term reintroduction program was initiated in an attempt to establish a self-sustaining population in the wild. Although a detailed pedigree has been established and utilized for captive management, including choosing breeding pairs, the current population exhibits signs of inbreeding depression [18]. For example, the species suffers from low hatching success [22]. Until establishment of the long-read genome assembly described here, molecular genetic studies were limited to small numbers of traditional genetic markers (e.g., microsatellite loci, amplified fragment length polymorphism; AFLP, and mitochondrial DNA markers [23,24]). These studies identified extremely low genetic diversity, which suggested that conservation efforts would benefit from a whole-genome approach that could generate resources for assessing the remaining polymorphic regions (e.g., SNPs, and structural variation).

In this study, we highlight the quality of the ʻAlalā genome assembly and compare it to other avian assemblies that were also generated from whole-genome shotgun-sequencing approaches. We provide details for a subset of candidate immunity genes that we hypothesize will have important conservation implications, and we examine the repeat composition of the genome. We also describe analyses of runs of homozygosity (ROH) and the fraction of the genome estimated to be completely autozygous (fROH [25]; i.e., identical by descent). Finally, we briefly discuss the goals and perceived challenges for the next stages of data generation and applications to ʻAlalā conservation and recovery.

## 2. Materials and Methods

### 2.1. Library Construction and Sequencing

Phenol-chloroform was used to extract high molecular weight genomic DNA from a blood sample taken from a single male ʻAlalā, named Hōʻike i ka pono (Figure S1, studbook #32). This individual was chosen for genome sequencing because (1) His high inbreeding coefficient (0.25) would allow for simplified genome assembly; and (2) He is a great-grandson of the two genetic founders that constitute approximately 45% of the ancestry in the captive population (i.e., his genome would be a good representation for most birds in the population [22]). Note that all procedures on live animals were approved by the Institutional Animal Care and Use Committee (IACUC) of San Diego Zoo Global (15-012 and 16-009). Library construction protocol followed the workflow for ultra-large insert libraries [26]. The DNA was sheared to target 50 kb fragments (resulting distribution 30–80 kb) by using a Megaruptor (Diagenode, Denville, NJ, USA), and assessed for quality by pulsed-field gel electrophoresis (PFGE) on the CHEF Mapper system (Bio-Rad, Hercules, CA, USA). A total of 86 µg of DNA were then recovered from the 50 kb shearing condition. Sheared DNA was constructed into SMRTbell templates (PacBio, Menlo Park, CA, USA) by following the >30 kb library construction protocol [26] with minor modifications (e.g., 1× AMPure PB purification (Beckman Coulter, Brea, CA, USA); room temperature rotation instead of vortexing; two-step elution process during AMPure PB elution to maximize recovery). Final SMRTbell library qualities were assessed by PFGE and Pippin Pulse (Sage Science, Beverly, MA, USA) to determine the optimal size-selection cut-off of 20 kb. Size selection was done using the BluePippin system (Sage Science), with targeted exclusion of small fragments (<20 kb) that would otherwise preferentially load during sequencing. Following size selection, the library fragments had a mode size of approximately 30 kb and comprised approximately 8.6 µg of DNA; enough to sequence 133 single-molecule, real-time (SMRT) cells at Pacific Biosciences (PacBio). Sequence data were generated using the PacBio RSII instrument with P6v2 polymerase binding, C4 chemistry kits (P6-C4) and 6 h run time movies, which yielded 9,859,413 reads, totaling 128,622,819,749 bp whole-genome sequence data. The average read length was 13,045 bp (max = 78,477 bp; standard deviation = 8972 bp). Reads less than 500 bp (1.7% of reads; <1% of total bases) were removed. Post-filtering, the N50 subread length was 18,661 bp.

### 2.2. Genome Assembly and Quality

*De novo* assembly followed the PacBio string graph assembler process, using FALCON and FALCON-Unzip [27] to generate long-range phased haplotypes. During the assembly process, sequence reads were overlapped to form long consensus sequences [6,27]. These longer reads were used to generate a string graph, and the graph was reduced so that multiple edges formed by heterozygous structural variation were replaced to represent a single haplotype [28]. Primary contigs were formed by using the sequences of non-branching paths, while associated contigs (i.e., haplotigs) represent the sequences of branching paths. The resulting assembly thus represents a phased diploid genome [27,29]. Primary and secondary genome assemblies are available on GenBank, Accession: QORP00000000. Raw reads are available at https://www.ncbi.nlm.nih.gov/sra/SRP151284.

To assess the quality of the final assembly, we compared the number and length of ʻAlalā contigs to those of other avian assemblies. In addition, we used BUSCO v2.0.1 [30] to assess the completeness of the gene space in the ʻAlalā assembly based on the detection of conserved single-copy orthologs. For comparison, we included genome assemblies of the domestic chicken (*Gallus gallus*, GenBank accession GCF_000002315.4 [31]), Anna’s hummingbird (*Calypte anna*, GCA_002021895.1 [29]), zebra finch (*Taenopygia guttata*, GCF_000151805.1 [32]), hooded crow (*C. cornix cornix*, GCF_000738735.1 [33]), and American crow (*C. brachyrhynchos*, GCF_000691975.1 [34]). As lineage datasets, we chose eukaryota_odb9 (303 genes) and a 250-gene eukaryotic subset [35], which is highly congruent with the core eukaryotic genes mapping approach (CEGMA) dataset [36]. Gene finding parameters in the AUGUSTUS analysis step were based on the chicken genome.

### 2.3. Repeat Composition Analysis

To identify mobile and repetitive DNA in the ʻAlalā assembly, we generated a *de novo* repeat library using RepeatModeler v1.0.11 [37]. This software package primarily integrates RECON v1.08 [38] and RepeatScout v1.0.5 [39] to find interspersed repeats. Repeat models with 50% sequence identity over at least half their length to Swiss-Prot entries with known function were removed from the library, and remaining models were assigned to repeat classes by reference to Repbase [40]. Additional, more detailed repeat classification was performed with CENSOR [41]. The ʻAlalā assembly was then screened for repetitive DNA using RepeatMasker v4.0.7 [37] based on RMBlast and two repeat libraries: (1) The ʻAlalā repeat library described above; and (2) An expanded library also containing all chicken and ancestral eukaryotic repeats, as well as all zebra finch repeats provided by Repbase. In addition to the version implemented in RepeatMasker, simple repeats were assessed using the stand alone version of Tandem Repeats Finder v4.0.9 [42] with the following settings: Match = 2, Mismatching penalty = 7, Delta = 7, PM = 80, PI = 10, Minscore = 50, and MaxPeriod = 2000. Along with the ʻAlalā assembly, we also analyzed the assemblies of the domestic chicken, Anna’s hummingbird zebra finch, hooded crow, and American crow listed above.

### 2.4. Candidate Gene Annotation and Analysis

We focused on annotating particular genes associated with adaptive and innate immunity, as diversity at such genes is predicted to be especially relevant to fitness. Specifically, we were interested in genes of the major histocompatibility complex class II beta (MHC class II B) and toll-like receptor (TLR) genes. To identify candidate immunity genes in the primary ʻAlalā assembly, we first performed Blast (tblastn) searches using homologous protein sequences of other bird species as queries, with an e-value cut-off of 1 × 10e^–5^. For MHC, queries were obtained from the zebra finch, the currently best annotated passerine genome [43] (Table S1). For the more conserved TLRs, the full gene repertoire of the domestic chicken was used (Table S1). In the absence of transcriptional evidence, individual ʻAlalā genes were located by comparing genomic coordinates of high-scoring segment pairs on each contig, which often corresponded to exons. Genomic sequence including 1500 bp up- and downstream of each putative gene was extracted, and the gene structure and coding sequence were predicted by the AUGUSTUS web server v3.3 [44]. In the case of MHC class II B, only putative genes including exons 2 and 3 were considered for this step, due to a large number of single-exon or fragmentary hits. Portions of nucleotides that appeared to be missing from the predicted coding sequence were identified by aligning the predicted sequence to the reference using MAFFT v7 [45]. Using short sequence motifs taken from the reference, we then attempted to find missing homologous parts by translating the genomic sequence into all three reading frames in the coding direction by using EMBOSS Transeq [46]. Finally, manually completed gene predictions were tentatively classified as functional, ambiguous or pseudogenized, depending on the integrity and length of the reading frame. Genes for which a complete reading frame, including start and stop codons, could be identified were considered functional, while genes that required the insertion or deletion of a single nucleotide to recover the complete reading frame (suggestive of a sequencing error) were categorized as ambiguous. Fragmentary reading frames or multiple frameshift mutations were regarded as indicative of pseudogenes. Untranslated 5′ and 3′ regions could not be annotated due to the lack of transcriptional evidence. MHC class II B Blast searches were later repeated to assess the number and divergence of gene fragments with increased sensitivity. Exons 2 and 3 of the ʻAlalā gene, Coha_MHCIIB_b (see Results), were used as query sequences.

To shed light on the evolutionary history of the gene family, we performed a phylogenetic analysis based on exon 2 of MHC class II B genes in ʻAlalā and other corvids. We included all functional and ambiguous ʻAlalā genes, as well as complete MHC class II B gene sets of single individuals, each of American crow (13 sequences), the jungle crow (*C. macrorhynchos japonensis*; 14 sequences), the Asian rook (*C. frugilegus*; 11 sequences) and the azure-winged magpie (*Cyanopica cyanus*, 7 sequences), to allow for within-genome diversity comparisons. These data were obtained by [47] using a targeted polymerase chain reaction (PCR) approach. Exon 2 nucleotide sequences were aligned with MAFFT v7, and 10 maximum likelihood trees computed under the GTRCAT model in RAxML v8.1.20 [48]. Confidence values were estimated from 500 rapid bootstrap replicates and drawn onto the best maximum likelihood tree (-f a algorithm).

### 2.5. MHC Functional Supertypes

To assess how similar the ʻAlalā MHC class II B repertoire might be to other corvids in terms of properties of the antigen-binding regions, we relied on comparisons of functional supertypes (e.g., [49,50]). Briefly, functional supertype analysis involves identifying codons under selection (positively-selected sites; PSS), and then grouping sequences according to descriptor variables that reflect the physical and chemical properties of the amino acids at these selected positions [51,52,53]. As the ʻAlalā MHC class II B sequences from this study were generated from a single individual, we based our analysis on the locations of nine PSS shared among three other crow species (jungle crow, carrion crow (*C. corone orientalis*), and American crow), which were previously identified through HYPHY analysis [54]. First, we used MUSCLE [55,56] implemented in Geneious vR10 [57] to align our putatively functional ʻAlalā exon 2 sequences to the 237 sequences from Eimes et al. [54], for a total of 244 nucleotide sequences. We then trimmed all sequences to exclude non-PSS codons, translated them, removed duplicate sequences (sequences remaining: 153), and converted the information into a matrix of five physiochemical descriptor variables that reflect the physical and chemical properties of each amino acid [51,52,53]: z1 (hydrophobicity), z2 (steric bulk), z3 (polarity), z4 and z5 (electronic effects). Using the matrix of z-descriptors, we performed *k*-means clustering with the adegenet package in R [58] to reveal clusters of sequences likely to have similar functional properties. We then used discriminant analysis of principle components (DAPC) to describe the clusters [59].

### 2.6. Runs of Homozygosity

Runs of homozygosity (ROH) are stretches of identical haplotypes that occur across homologous chromosomes within the same individual. The length of ROH segments within an individual’s genome depends on whether shared ancestry is recent or ancient; recent inbreeding results in relatively long ROH segments, because recombination has not yet broken up the segments that are identical by descent [60]. As mutations accumulate over time, the ROH segments further break down. We assessed ROH in the ʻAlalā genome for three purposes: (1) To estimate the autozygous fraction of the genome fROH [25], i.e., the total fraction of the genome that is perfectly autozygous (zero heterozygosity); (2) To evaluate the effect of allowance for low levels of heterozygosity on estimates of whole-genome fROH and ROH segment lengths; and (3) To estimate fROH on a per-contig basis. For comparison to an outbred species, the fROH and ROH segment length analyses were also conducted using an Anna’s hummingbird genome that was sequenced and assembled similarly to the ʻAlalā genome assembly [29]. The ROH segment lengths were calculated by summing consecutive sliding windows that met criteria of perfect autozygosity or fell within the one of four heterozygosity thresholds: 1 SNP per 100 kb, 50 kb, 25 kb or 10 kb (i.e., ≤0.01, ≤0.02, ≤0.04, and ≤0.1 SNPs/kb). The fROH was calculated by taking the sum of ROH segment lengths and dividing this number by the cumulative length of all sliding windows. The sliding windows, set to 100 kb, were calculated with vcftools [61] using SNPs called from the primary contigs. Only contigs ≥500 kb were included in this analysis, permitting a minimum of five consecutive sliding windows to assess ROH segment lengths. As sliding window analysis incorporates rounding, the cumulative length of sliding windows exceeds the cumulative length of contigs.

## 3. Results

### 3.1. Genome Assembly and Quality

**Figure 1:**
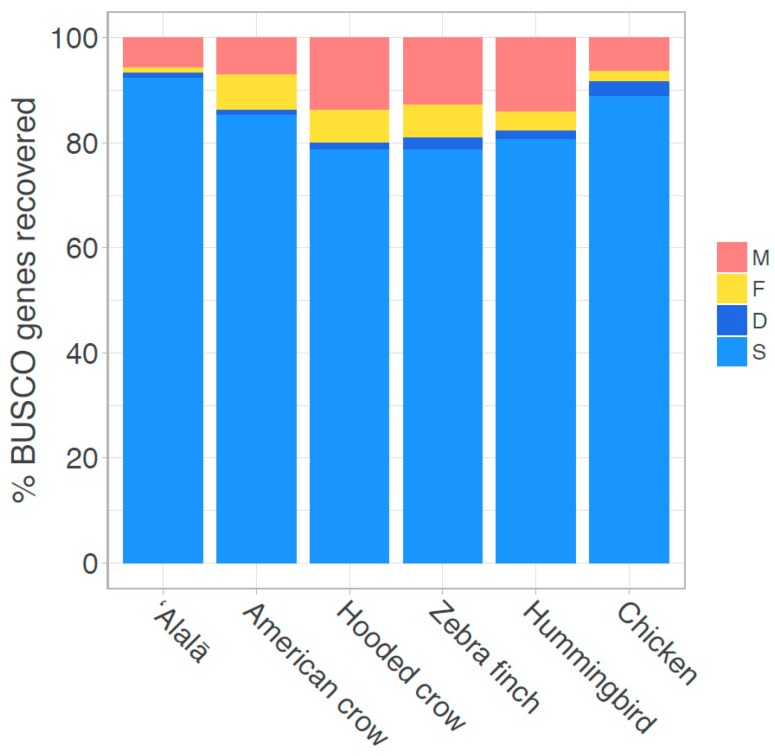
Genome assembly completeness assessed by the recovery of universal single-copy genes (BUSCOs). Percentages refer to complete genes that were found as single (S) or multiple copies (D), as well as fragmented (F) and missing (M) genes. Analyses were based on the BUSCO eukaryote dataset (*n* = 303 genes).

The FALCON assembler generated a 1.06 Gbp primary assembly with a contig N50 of 7,737,654 bp across 671 total contigs (Table 1). The diploid assembly process produced 2082 associated haplotype contigs (haplotigs) with an estimated length of 0.43 Gb and contig N50 of 455,082 bp (Table 1), implying that about 40% of the genome contained sufficient heterozygosity to be phased into haplotypes by FALCON-Unzip. For comparison, the same assembly process suggested that 75% and 100% of the genomes of two more outbred species, zebra finch and Anna’s hummingbird, contained sufficient heterozygosity to be phased into haplotypes [29] (Table 1). Compared to other published short-read based avian genomes of similar size, the ʻAlalā assembly represents a dramatic decrease in assembly fragmentation, with substantially fewer and longer contigs, and is similar in quality to other long-read *de novo* assemblies (Figure 1; Table S2). The BUSCO analysis indicated that gene completeness was among the highest of any avian genome to date (Figure 1 and Table S2). Collectively, these results suggest that this ʻAlalā long-read genome assembly is one of the highest quality avian genomes currently available.

### 3.2. Mobile and Repetitive Elements

*De novo* repeat modeling resulted in an ʻAlalā-specific repeat library containing 260 families, including 50 LINE (long interspersed element) and 23 LTR (long terminal repeat) families. Only 12% of these had matching entries in Repbase, mostly to endogenous retroviruses (ERVs) and CR1 retrotransposons previously identified in other passerine birds. In addition, several ʻAlalā repeat families were partially similar to the large tandem repeat ʻcrowSat1’, a 14 kb satellite that is suspected to be a major heterochromatin component in the hooded crow [62]. In contrast, extended matches to Swiss-Prot entries [63], which might indicate co-opted transposable elements [64], were not discovered. RepeatMasker screening identified 10.1% of the ʻAlalā assembly as mobile or repetitive sequence, including 3.3% LINEs (exclusively of the CR1 class), 1.1% LTRs (various endogenous retroviruses), and 4.5% unclassified interspersed repeats. The remaining 1.2% was made up of simple repeats and low complexity sequence, including satellites homologous to crowSat1. This estimate did not change noticeably when using a repeat library expanded with avian and ancestral eukaryotic repeats provided by Repbase. The stand-alone analysis of Tandem Repeats Finder revealed 303,030 tandem repeats with a maximum unit length of 2000 bp, making up 6.9% of the assembly (max. repeat size was ~100 kb). This fraction is substantially lower than in the domestic chicken (16.1%), but higher than in the zebra finch (3.7%), Anna’s hummingbird (3.1%), the American crow (2.8%), and the hooded crow (1.8%). However, these results might partially reflect differences in assembly completeness and contiguity, which affect repeat identification (e.g., the American crow and hooded crow genome assemblies are short-read based; the Anna’s hummingbird genome was generated and assembled using a similar process as for the ʻAlalā). When combined, the RepeatMasker and Tandem Repeats Finder estimates suggest that the ʻAlalā assembly contains approximately 15% repetitive DNA. Since heterochromatic regions often cannot be assembled reliably, this is likely an underestimate of the true genome repeat content.

### 3.3. Candidate Gene Annotation and Analysis

We identified eleven (including duplicates) TLR genes in the ʻAlalā genome (Table S3). Sequence and gene structure, which could be reliably assessed with only a single untranslated 5′ exon missing from one prediction, were highly conserved with regard to the chicken reference [65] and other birds [66]. All genes were classified as functional based on possessing complete or nearly complete open reading frames. Notable features relative to the reference include the loss of 140 amino acids at the 5′ end in *TLR1A*, a ~50 amino acid indel in *TLR2B*, and a tandem arrangement of two *TLR7* copies differing by 16 amino acids. This duplication has previously been observed in other passerine birds [66,67,68,69,70] and was annotated here as *TLR7a* and *TLR7b*.

The MHC class II B repertoire of the ʻAlalā proved to be more complex. We identified seven presumably functional and two ambiguous, but in all likelihood equally functional, genes with open reading frames across all five expected exons in the primary assembly. Additionally, we discovered three genes with incomplete or disrupted reading frames comprising exons 2 and 3 (Table S4). Most of these genes were located in tandem arrays of 20–40 kb (b-c-d, e-f-g, and h-i-p1). Uniform read coverage, small but detectable differences in flanking regions, and reads spanning multiple genes suggest that these genes represent individual loci, rather than alleles or assembly artifacts. Overall, the ʻAlalā MHC class II B genes proved largely conserved compared to the zebra finch reference (sequence identity > 80% at the amino acid level), with the highest variability found in exon 2 as expected. In addition to the three more complete putative pseudogenes above, the assembly also contains a large number of MHC class II B fragments. More than 130 sequences homologous to exon 2, and about 30 homologous of exon 3 (containing the immunoglobulin C1-set domain) were found scattered throughout the primary assembly, usually in the form of tandem arrays of 2–5 copies (Table S5). According to phylogenetic analysis, six of the functional and ambiguous ʻAlalā MHC class II B genes comprise a strongly supported clade (Figure S2). These copies only differ by 0–3 amino acids, which were found at or very close to the positively selected sites (PSS) described for other corvids [54]. In contrast, only three genes—highly similar copies ʻa’ and ʻd’, as well as copy ʻg’—were placed in other locations of the phylogenetic tree, albeit with lower support.

### 3.4. MHC Functional Supertypes

**Figure 2:**
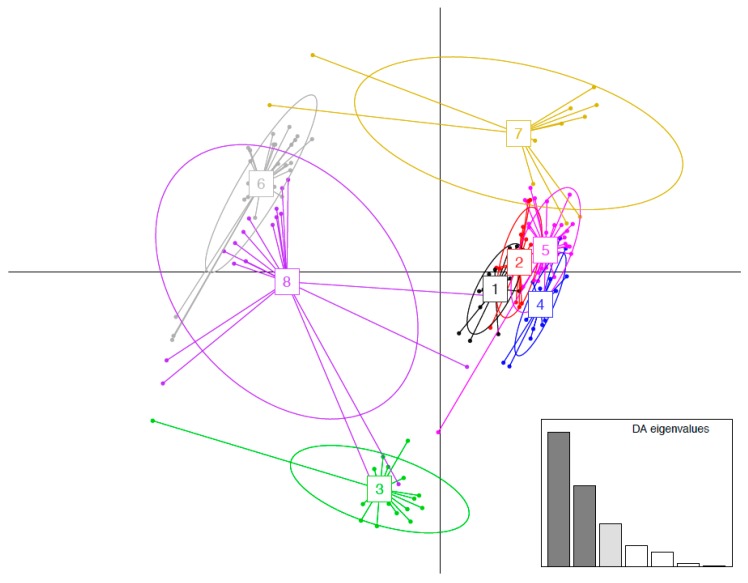
Discriminant Analysis of Principle Components (DAPC) scatterplot of the 8 major histocompatibility complex (MHC) supertypes identified here. 10 principle components (PC) and three discriminant functions (dimensions) were used to describe the relationship between the clusters. The scatterplot show only the first two discriminant functions (*d* = 2). The bottom graph displays the barplot of eigenvalues for the discriminant analysis (DA). Dark grey, light grey and white bars indicate eigenvalues that were used in the scatterplot, not used in the scatterplot but retained for the analysis, and not retained for the analysis, respectively. Each allele is represented as a dot, and the supertypes as ellipses.

**Figure 3:**
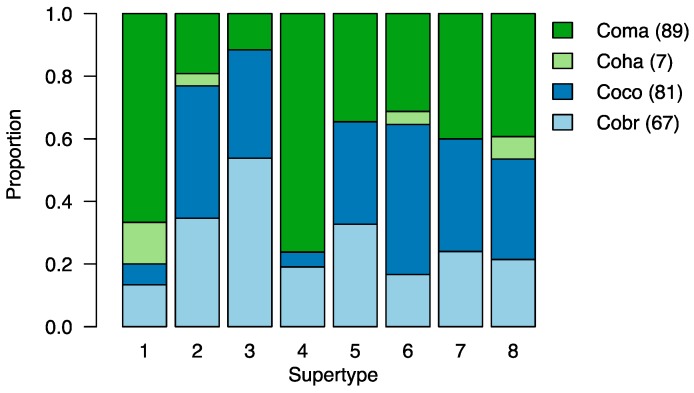
Stacked barplot indicating the representation of each corvid species within each MHC supertype identified here. The three other corvid species were represented across all eight supertypes, while the ʻAlalā was represented by three supertypes. Note, however, that the ʻAlalā data were established from a single individual, while the other species’ data represent 4–6 individuals per species [54]. In the legend: Coma = jungle crow; Coha = ʻAlalā; Coco = carrion crow; Cobr = American crow; Numbers in brackets indicate the number of nucleotide sequences included in the analysis.

Similarity of the ʻAlalā MHC class II B repertoire in terms of functional antigen-binding properties to three other corvids was assessed on the basis of nine PSS [54]. Focusing on these PSS in 244 nucleotide sequences, we identified 153 unique amino acid variants. From these, we identified eight functional supertypes (Figure 2), consistent with [54]. Three of the eight supertypes corresponded to ʻAlalā and were also shared by the three other corvid species that were compared (Figure 3). No ʻAlalā supertypes were discovered to be separate from other corvids. It must be noted, however, that sequences from only one ʻAlalā were used (compared to 4–6 individuals for each of the other species included in the supertype analysis). Our purpose in performing this supertype analysis was not to fully characterize the repertoire of MHC class II B diversity or functionality in ʻAlalā, but rather to establish a sense of how similar (or different) these *might* be compared to related species. The information gained has been used to design primers for targeted-amplicon approaches that are now being used to better assess ʻAlalā MHC class II B diversity at the population level.

### 3.5. Runs of Homozygosity

A total of 413,114 SNPs were detected across the 209 ʻAlalā genome contigs >500 kb in length (1.02 Gb of sequence data, 96% of the genome). Based on sliding windows intervals of 100 kb, the fraction of the genome that was perfectly autozygous (i.e., fROH) was approximately 5.6% (574 of 10,338 sliding windows were completely homozygous, Table S6). Allowing for ROH to include low level heterozygosity resulted in a substantial increase in the number of ROH segments and fROH. For example, with a heterozygosity threshold of ≤1 SNP per 50 kb, the number of sliding windows counted as ROH increased to 2496 and fROH increased to 46.1% (Table S6). With regard to ROH segment lengths, the ROH tended to be short under the criterion of perfect autozygosity, but increased in length as the allowable heterozygosity threshold was raised. In terms of individual contigs, the proportion of perfectly autozygous ROH relative to all sliding windows was highly variable and ranged from a minimum of 0% to a maximum of 63% (median 2.7%; average 5.7%). For the hummingbird, 1,841,031 SNPs were detected across 283 genome contigs >500 kb in length (0.92 Gb of sequence data, 92% of the genome). The fraction of the genome that was perfectly autozygous was 4.4% (416 of 9373 sliding windows, Table S6), fairly similar to that of the ʻAlalā. However, in contrast to the ʻAlalā, allowing for low levels of heterozygosity had little impact on the number of ROH segments and fROH. For example, raising the allowable heterozygosity threshold to ≤1 SNP per 50 kb only increased the number of sliding windows counted as ROH to 557 and fROH to 5.9%. The ʻAlalā’s sensitivity to allowing for low-levels of heterozygosity in ROH calculations is explained by a general trend for low genetic diversity across most of its genome, with median and mean values of 0.05 and 0.40 SNP/kb across the 100 kb sliding windows. For comparison, hummingbird median and mean values were 1.93 and 1.96 SNP/kb, which explains why the heterozygosity threshold had little effect on ROH and fROH calculations.

## 4. Discussion

Using PacBio SMRT sequencing technology and FALCON assembly, we generated a high-quality, long-read *de novo* genome assembly for one of the world’s most endangered birds. During the assembly process, FALCON stipulates that if overlapping regions differ by ≥5% over extensive distances then the assembler separates the regions into primary and associated (secondary) contigs [29]. By definition, regions of the primary assembly that have corresponding associated contigs identify areas in the genome with relatively high heterozygosity. The genome assembly of a single ʻAlalā (studbook #32, Figure S1) highlights the genomic signatures of small population size and inbreeding, because the ʻAlalā associated contigs corresponded to a substantially smaller proportion of the genome compared to more outbred species.

### 4.1. Candidate Immunity Genes

Toll-like receptors are an integral component of the innate immune system in animals. Recognizing pathogen-associated molecular patterns (PAMPs) at the cell surface, their role consists of activating the organism’s inflammatory response through a cascade of intracellular signals. Because variation in TLRs is associated with resistance or susceptibility to infectious diseases, the gene family is especially relevant regarding fitness and inbreeding-related conservation matters. In the ʻAlalā, we discovered a full complement of functional TLR genes (Table S3), with high conservation of gene number, structure, and sequence in comparison to other birds [62]. Although only a single *TLR7* copy exists in most other birds with annotated TLR repertoires, including the zebra and house finch, duplicates have been reported in several passerine species [66,67,68,69,71], indicating that duplication likely predates the split of the corvid family from other passerines. We did not find evidence that *TLR5* was pseudogenized in ʻAlalā, as it is in some passerine species [72]. However, as this result is based on a single individual it should be taken with caution.

In contrast to TLRs, the MHC, which activates the adaptive branch of the vertebrate immune system, includes some of the most variable genes found in vertebrates. MHC class II B genes, on which we focused in this study, encode proteins that can bind and present a large range of pathogen-derived peptides. Studying MHC diversity, particularly of the peptide-binding domain located on exon 2 (class II histocompatibility antigen B domain), can therefore be highly relevant for the conservation of endangered species. The number of functional MHC class II B genes we identified in the ʻAlalā (~7–9; Table S4) places it within the range known from other corvids (7–20 alleles per individual were described in the American crow, jungle crow, and carrion crow [54]). The MHC class II B diversity in the assembly appears to be low, with six out of nine putatively functional genes being almost identical at the amino acid level. Although we cannot completely rule out that some of these genes represent allelic variants of each other, evidence obtained from read coverage and flanking regions suggest that all genes are derived from genuine and separate loci. Several recent episodes of gene duplication may account for the pattern of high similarity, possibly including segmental duplications that gave rise to the observed tandem arrays with relatively high sequence homology up- and downstream of the MHC copies. According to our phylogenetic analysis, these events must have occurred after the divergence of the ʻAlalā from the other represented corvids (Figure S2). Alternatively, the true evolutionary history of the MHC class II B gene family may have become obscured by gene conversion, which can homogenize gene copies within a species, and has been implicated in other birds [73,74]. Intronic and exon 3 sequences from additional corvids and ʻAlalā individuals could shed light on this issue in the future (e.g., [73]). The three remaining ʻAlalā MHC class II B genes that did not fall within the main cluster, on the other hand, may represent remnants of evolutionarily distinct gene family lineages. However, a lack of support along the backbone of the phylogenetic tree prevented the identification of clear orthologs within other species. The phylogenetic analysis and previous studies [47,54] also revealed multiple MHC class II B lineages shared by other corvids, suggesting that the gene family expanded prior to the radiation of the corvid family. Notably, no ʻAlalā genes were found within several of these lineages consisting of genes from all or almost all other corvids investigated here (Figure S2). In all likelihood, these genes were lost in the evolutionary lineage leading to the ʻAlalā, or even very recently in the species’ population history. This hypothesis is supported by the high number of gene fragments and high sequence similarity between copy ʻg’ and pseudogene ʻp1’, suggesting at least one evolutionarily recent pseudogenization event. More generally, the large number of putative MHC class II B pseudogenes (Table S5) is consistent with expectations for passerines (e.g., [75]), and the evolution of a gene family characterized by repeated gene gain and loss. Most of these pseudogenes appeared to be highly fragmented, i.e., homology could only be established over a short length of exon 2 or 3 (150 bp or less). Sequence identity to the functional Coha_MHCIIB_b copy fluctuated widely, ranging from near perfect matches to less than 40% at the amino acid level, suggesting a broad age distribution with regard to the time of pseudogene origin. This might be a reflection of the dynamic evolution of this hyper-variable gene family, which included repeated expansions and contractions over evolutionary timescales [76]. The localization of most fragments on tandem arrays with high sequence homology in adjacent regions (alignment of 20 randomly chosen pseudogenes ±250 bp up- and downstream) also suggests that frequent segmental duplication events contributed to the abundance of MHC class II B pseudogenes. What remains unclear is why most pseudogenes seem limited to fragments of exons 2 or 3, rather than full-length genes including more conserved exons. A few fragments, especially those on shorter contigs, may represent assembly artifacts. Another possibility is that some are functional parts of other genes that originated by exon-shuffling. Further improvements to the assembly and a comprehensive annotation of the entire ʻAlalā gene content may bring more clarity on this matter in the future.

In summary, the present long-read ʻAlalā genome assembly includes more complete gene sequences than are available for many avian genomes, a crucial factor for annotating complex genomic regions, such as the MHC. While the observations described here are based on only a single individual, and should thus be interpreted with caution, our results imply that MHC class II B diversity in the ʻAlalā is likely to be somewhat similar by comparison to other corvids. Additionally, our functional supertype analysis suggests that while nucleotide sequences may differ between ʻAlalā and other corvids, similarity exists among species when it comes to pathogen-binding properties. However, genome data from additional specimens are required to gauge within-species diversity and distribution of different MHC class II B lineages, which would further place these data into the context of corvid and ʻAlalā evolution.

### 4.2. Runs of Homozygosity

If SNPs were evenly distributed across the genome, the SNP encounter rate in ʻAlalā would be 1 SNP per 2477 bp (413,114 SNPs identified from 1.02 Gb of sequence data). This value is considerably lower than empirical estimates obtained from genomes of other avian species, for example, 1 SNP per 330 bp in *Ficedula* flycatchers [77], 1 SNP per 256 bp in Hawaiʻi Amakihi (*Hemignathus virens* [78]), 1 SNP per 935 bp in turkey (*Meleagris* spp. [79]), and 1 SNP per 501 bp in Anna’s hummingbird [29] (based on contigs ≥500 kb). While caution should be used when making comparisons between genomes that differ by sequencing technologies, genome assembly pipelines, and other computational settings (addressed in more detail below), the paucity of SNPs in the ʻAlalā genome is not surprising because of the overall low population size of ʻAlalā and this particular bird’s high pedigree inbreeding coefficient (0.25). By comparison to the examples noted here, the ʻAlalā genome was generated and assembled in a similar fashion to that of the Anna’s hummingbird, the latter of which had a SNP encounter rate almost five times as frequent. Certainly, the presence of contigs showing very low heterozygosity in the ʻAlalā is consistent with empirical observations made of ROHs in turkey [79] and large stretches of very low heterozygosity in Hawaiʻi Amakihi [78]. The contrast between highly variable sliding windows and regions with modest variability suggests that the PacBio assembly pipeline used here is sensitive to calling SNPs across a range of heterozygosities, and that low diversity observed for this genome is not solely an artifact of the assembly pipeline.

The lengths of ROH are an indication of shared ancestry and can be used to gauge whether inbreeding events occurred within recent or distant generations [60]. Recombination events break long autozygous segments into smaller pieces, thus numerous short ROH are consistent with distant shared ancestry. In this ʻAlalā genome, the numerous short ROH collapse into much longer segments when low levels of heterozygosity are allowed. This indicates both mutations and recombination have a strong impact on ROH measures in the ʻAlalā. In contrast, the hummingbird had far fewer ROH, and relaxing the heterozygosity threshold had relatively less impact on ROH numbers and lengths. Thus, recombination, not mutations, was the dominant force in ROH segment lengths in the hummingbird. This comparison between two species that differ by demographic histories highlights the sensitivity of ROH to patterns of mutations in the genome, along with SNP filtering criteria. Moreover, the rate of sequencing error (related to depth of sequencing and sequencing platform) will also affect the estimations of homozygosity and, correspondingly, the length of ROH segments.

Several factors confound comparability of ROH across studies and taxa. These include: Lack of consensus definition for ROH; differences in sequencing platforms and associated sequencing errors; variant-calling pipeline; and computational settings (e.g., [80,81,82]). The ROH estimates in this study are drawn from a single genome with high depth of sequencing. In contrast, measures of ROH can be obtained from high-density SNP arrays by quantifying the length of rows of homozygous SNPs relative to a reference, the results of which are sensitive to SNP chip density, and may miss unmeasured variants between the markers [80,81]. The density of SNP markers across the genome also impacts what can be reliably detected as a minimum length ROH. For example, in humans, a panel of 3 million SNP markers identified ROH as short as 100 kb, and by applying this denser SNP panel to the Han Chinese population, their estimated total ROH increased from 130 Mb, measured with a 0.4 million SNP panel, to 510 Mb [60]. Comparability of ROH results between species can be further diminished by biological variability in chromosome lengths. Birds, with numerous microchromosomes, are expected to have shorter ROH than mammals, simply because of differences in chromosome lengths.

### 4.3. Applications to ʻAlalā Conservation

The ʻAlalā genome assembly resulting from long-read sequencing data provides a high-quality reference genome that will enable downstream comparative, population, and conservation applications. Prior to this study, molecular work using limited genetic markers identified low diversity in the species [23,24], and pedigree analysis identified inbreeding effects on hatching success, as well as skewed founder representation [18,22]. Thus, we identified a need to generate genomic resources, particularly to inform strategic pairings in an effort to slow the rate of inbreeding (and increase the population growth rate) and to preserve remaining genetic diversity in order to maximize the likelihood that the species will be able to adapt to environmental changes. Work is also ongoing to better understand the fitness associations of particular candidate genes; for example whether these, in addition to genome-wide diversity, are linked to hatching success. Similarly, identifying the genetic basis of other fitness-related phenotypes is a critical research goal that could be incorporated with strategic pairings. For example, the California condor (*Gymnogyps californianus*) has a relatively high frequency of heritable, embryonic lethal dwarfism, and genomics are currently being applied to identify carriers of the disease in an effort to eliminate it from managed populations [83,84,85,86,87]. Using genomic information, a similar strategy could be applied to managing maladaptive phenotypes in ʻAlalā. 

Genomic data derived from our analyses are an essential component of the current and future recovery of the ʻAlalā. The use of genomic information to assist strategic pairing and minimize inbreeding is the most practical and immediate application of genomics at this time, and is predicted to have a positive impact on population growth (e.g., [22], but also see [88]). In conjunction with ongoing conservation-breeding activities, a reintroduction program was recently initiated in an effort to re-establish this formerly extinct-in-the-wild species into its native forest habitat. Early indications of the reintroduction effort are promising, with a small population of recently released individuals surviving in the wild at the time of writing. Because the entire extant ʻAlalā population, both in captivity and the wild, is still relatively small, ongoing management decisions for the breeding and release of particular individuals will have implications for the long-term recovery of the species. As the size of both the captive and wild ʻAlalā populations continue to increase, the integration of genomic data as part of the conservation management effort will help to maximize the genetic health of the species well into the future. 

## Figures and Tables

**Table 1:** *De novo* long-read genome assembly statistics comparing PacBio-based primary and secondary haplotypes in three avian species.

Species	PacBio-Based Primary Haplotype	PacBio-Based Secondary Haplotype
**ʻAlalā** (this study)		
Number of contigs	671	2082
Contig N50	7,737,654 bp	455,082 bp
Total size	1,064,991,496 bp	432,637,353 bp
**Zebra finch** [29]		
Number of contigs	1159	2188
Contig N50	5,807,022 bp	2,740,176 bp
Total size	1,138,770,338 bp	843,915,757 bp
**Anna’s hummingbird** [29]		
Number of contigs	1076	4895
Contig N50	5,366,327 bp	1,073,631 bp
Total size	1,007,374,986 bp	1,013,746,550 bp

## Supplementary Materials

The following are available online at http://www.mdpi.com/2073-4425/9/8/393/s1. References [89,90,91,92,93,94,95,96,97] are cited in the Supplementary Materials. Table S1. Accessions of references used to annotate candidate immunity genes. Table S2. ʻAlalā assembly results compared to other avian genome assemblies. Statistics are given for contigs, and for scaffolds in parentheses. Table S3. ʻAlalā toll-like receptor (TLR) genes, following the nomenclature suggested by [65]. Start and stop refer to the position of the start and stop codons on the contig. Exons indicates the number of exons identified in the absence of transcriptional evidence, with the number of exons in the *G. gallus* reference given in parentheses. All predicted genes appear to be complete, suggesting they are functional (F). Notes are provided with respect to the *G. gallus* reference, where applicable. aa: amino acids. Table S5. Homologs of MHC class II B exons 2 and 3 in the ʻAlalā genome. Identity (% id), mismatches (Mism.), gaps, query start and end positions (Q-start, Q-end), hit start and end positions (H-start, H-end), e-value and score refer to Blast results using Coha_MHCIIB_b exons 2 and 3 as query. Bit scores > 100 are highlighted in yellow. Consecutive hits on the same contig within 1500 bp (in green blocks) were annotated further as MHC class II B candidate genes (see Table S3). The remaining hits are fragments that may represent, pseudogenized copies. Table S6. Analysis of runs of homozygosity (ROH) for Alala (*Corvus hawaiiensis*, Coha) and Anna’s hummingbird (*Calypte anna*, Caan) [29] genome contigs of minimum length 500 kb (*n* = 209 and *n* = 283, respectively). The ROH segment length is the cumulative length of uninterrupted 100 kb sliding windows (SW) that were completely autozygous or fell within one of four heterozygosity thresholds. The autozygous fraction of the genome (fROH) is calculated as the total number of sliding windows in ROHs (# SW-ROH) divided by the total number of sliding windows (*n* = 10,338 Coha; *n* = 9373 Caan) across all genome contigs. Figure S1. ʻAlalā pedigree. The sequenced individual, studbook 32 (named Hōʻike i ka pono) is shaded. Dashed lines indicate individuals that are represented in multiple positions in the pedigree (e.g., overlapping generations). Figure S2. Unrooted maximum likelihood tree of corvid MHC class II B genes, exon 2. Functional genes predicted from the ʻAlalā assembly are highlighted in red (prefix Coha). Other species represented include *C. brachyrhynchos* (American crow, Cobr), *C. macrorhynchos* (jungle crow, Coma), *C. frugilegus* (Asian rook, Cofr) and *Cyanopica cyanus* (azure-winged magpie, Cycy). All genes were obtained from one individual per species [47]. Confidence values are given for nodes with bootstrap support >70%.
