# Supplementary material for: A High-Quality, Long-Read De Novo Genome Assembly to Aid Conservation of Hawaiiʻs Last Remaining Crow Species
Source: Genes (Basel). 2018 Aug 1;9(8):393. doi: 10.3390/genes9080393 (PMC6115840; doi:10.3390/genes9080393)
Supplement: Supplementary file 1 [file genes-09-00393-s001.pdf]

genes

MDPI

*Supplementary Materials*

# A High-Quality, Long-Read *De Novo* Genome Assembly to Aid Conservation of Hawaii's Last Remaining Crow Species

**Jolene T. Sutton, Martin Helmkampf, Cynthia C. Steiner, M. Renee Bellinger, Jonas Korlach, Richard Hall, Primo Baybayan, Jill Muehling, Jenny Gu, Sarah Kingan, Bryce M. Masuda and Oliver A. Ryder**

**Table S1**. Accessions of references used to annotate candidate immunity genes.

| Gene group | Reference species | Reference accession | Source |
|---|---|---|---|
| MHC | zebra finch | UniProtKB: H1A2M7_TAEGU | [32] |
| | | UniProtKB: H0ZVH6_TAEGU | [32] |
| | | UniProtKB: H0ZU54_TAEGU | [32] |
| | | UniProtKB: H1A3G3_TAEGU | [32] |
| | | UniProtKB: H0ZXA5_TAEGU | [32] |
| | | UniProtKB: H0ZZB1_TAEGU | [32] |
| | | UniProtKB: H1A0J1_TAEGU | [32] |
| | | UniProtKB: H0ZWA2_TAEGU | [32] |
| | | UniProtKB: H0ZWI5_TAEGU | [32] |
| | | UniProtKB: H0ZWL2_TAEGU | [32] |
| | | UniProtKB: H0ZXL7_TAEGU | [32] |
| TLR | chicken | GenBank: NM_001007488.4 | [89] |
| | | GenBank: NM_001081709.3 | [90] |
| | | GenBank: NM_204278.1 | [91] |
| | | GenBank: NM_001161650.1 | [92] |
| | | GenBank: NM_001011691.3 | [93] |
| | | GenBank: NM_001030693.1 | [94] |
| | | GenBank: NM_001024586.1 | [95] |
| | | GenBank: NM_001011688.2 | [96] |
| | | GenBank: NM_001037835.1 | [97] |
| | | GenBank: NM_001030558.1 | [94] |

**Table S2**. ʻAlalā assembly results compared to other avian genome assemblies. Statistics are given for contigs, and for scaffolds in parentheses.

| Assembly | Accession | Technology | # Bases | # Contigs | Largest | N50 |
|---|---|---|---|---|---|---|
| ʻAlalā (*C. hawaiiensis*) | (Primary contigs; this study) | PacBio SMRT | 1.06 Gb | 671 | 31.5 Mb | 7.7 Mb |
| American crow (*C. brachyrhynchos*) | GCF_000691975.1 | Illumina HiSeq | 1.05 Gb (1.09 Gb) | 89,646 (10,547) | 265 kb (25.9 Mb) | 29.1 kb (7.0 Mb) |
| Hooded crow (*C. cornix cornix*) | GCF_000738735.1 | Illumina HiSeq | 1.02 Gb (1.05 Gb) | 28,561 (1,299) | 1.3 Mb (50.2 Mb) | 96.4 kb (16.4 Mb) |
| Zebra finch (*Taeniopygia guttata*) | GCF_000151805.1 | Sanger | 1.22 Gb (1.23 Gb) | 124,806 (37,096) | 425 kb (156.4 Mb) | 38.6 kb (62.4 Mb) |
| Anna's hummingbird (*Calypte anna*) | GCA_002021895.1 | PacBio SMRT | 1.01 Gb | 1,076 | 25.8 Mb | 5.37 Mb |
| Domestic chicken (*Gallus gallus*) | GCF_000002315.4 | multiple | 1.22 Gb (1.23 Gb) | 24,695 (23,475) | 17.5 Mb (196.2 Mb) | 2.9 Mb (82.3 Mb) |

**Table S3**. ʻAlalā toll-like receptor (TLR) genes, following the nomenclature suggested by [65]. Start and stop refer to the position of the start and stop codons on the contig. Exons indicates the number of exons identified in the absence of transcriptional evidence, with the number of exons in the *G. gallus* reference given in parentheses. All predicted genes appear to be complete, suggesting they are functional (F). Notes are provided with respect to the *G. gallus* reference, where applicable. aa: amino acids.

| Gene | Contig | Start | Stop | Orient. | No. aa | Exons | Status | Notes |
|---|---|---|---|---|---|---|---|---|
| Coha_TLR1A | 000007F | 17637771 | 17640173 | + | 661 | 1 (2) | F | 5′ end 140 aa shorter |
| Coha_TLR1B | 000007F | 17626125 | 17628053 | + | 643 | 1 (1) | F | |
| Coha_TLR2A | 000039F | 7428319 | 7430634 | + | 772 | 1 (2) | F | |
| Coha_TLR2B | 000039F | 7435849 | 7438197 | + | 771 | 1 (2) | F | ~50 aa indel |
| Coha_TLR3 | 000007F | 8442693 | 8448953 | + | 896 | 4 (5) | F | |
| Coha_TLR4 | 000034F | 6443202 | 6446898 | – | 843 | 3 (3) | F | |
| Coha_TLR5 | 000035F | 2853399 | 2855912 | – | 838 | 1 (1) | F | |
| Coha_TLR7a | 000000F | 13894219 | 13897341 | – | 1027 | 1 (2) | F | tandem duplicate (diff. 16 aa) |
| Coha_TLR7b | 000000F | 13880285 | 13883407 | – | 1027 | 1 (2) | F | tandem duplicate (diff. 16 aa) |
| Coha_TLR15 | 000045F | 6705567 | 6708188 | – | 874 | 1 (1) | F | |
| Coha_TLR21 | 000253F | 183362 | 186423 | + | 968 | 2 (2) | F | |

**Table S4.** ʻAlalā MHC class II B genes. Beginning and end coordinates refer to initial, uncurated gene models, and may or may not coincide with start and stop codons. The Exons column provides the number of exons identified in the absence of transcriptional evidence. The Status column indicates whether the predicted gene is presumably functional (F), ambiguous (A) or a pseudogene (P) according to the definition given in the main text. Only pseudogenes containing both exon 2 and 3 are listed. "Frameshift fixed" denotes gene models that were fully recovered by the insertion of a single nucleotide at a homopolymer (suggesting a sequencing error).

| Gene | Contig | Begin | End | Orient. | No. aa | Exons | Status | Notes |
|---|---|---|---|---|---|---|---|---|
| Coha_MHCIIB_a | 000257F | 17839 | 19521 | – | 250 | 5 | F | |
| Coha_MHCIIB_b | 000357F | 54501 | 55649 | + | 250 | 5 | F | |
| Coha_MHCIIB_c | 000357F | 69097 | 70245 | + | 250 | 5 | F | |
| Coha_MHCIIB_d | 000357F | 80536 | 81696 | + | 250 | 5 | F | |
| Coha_MHCIIB_e | 000485F_001 | 9214 | 10361 | + | 250 | 5 | A | Frameshift fixed |
| Coha_MHCIIB_f | 000485F_001 | 29737 | 30884 | + | 250 | 5 | F | |
| Coha_MHCIIB_g | 000485F_001 | 46119 | 47225 | + | 250 | 5 | F | |
| Coha_MHCIIB_h | 000868F | 15490 | 16637 | + | 250 | 5 | F | |
| Coha_MHCIIB_i | 000868F | 26421 | 27564 | + | 249 | 5 | A | Frameshift fixed |
| Coha_MHCIIB_p1 | 000868F | 4152 | 5188 | + | 251 | 5 | P | Divergent, multiple frameshifts, non-canonical splice site |
| Coha_MHCIIB_p2 | 000251F | 76819 | 77558 | + | 212 | 3 | P | Fragmentary (exons 1–3), frameshift, mutated splice site |
| Coha_MHCIIB_p3 | 000219F | 136202 | 137011 | + | 99 | 3 | P | Fragmentary (exons 1–3), multiple frameshifts |

**Table S5**. Homologs of MHC class II B exons 2 and 3 in the ʻAlalā genome. Identity (% id), mismatches (Mism.), gaps, query start and end positions (Q-start, Q-end), hit start and end positions (H-start, H-end), e-value and score refer to Blast results using Coha_MHCIIB_b exons 2 and 3 as query. Bit scores > 100 are highlighted in yellow. Consecutive hits on the same contig within 1500 bp (in green blocks) were annotated further as MHC class II B candidate genes (see Table S3). The remaining hits are fragments that may represent, pseudogenized copies.

| Exon | Contig | %id | Length | Mism. | Gaps | Q-start | Q-end | H-start | H-end | E-value | Score |
|---|---|---|---|---|---|---|---|---|---|---|---|
| 2 | 000114F | 48 | 44 | 23 | 0 | 41 | 84 | 3271249 | 3271118 | 3.00E-07 | 53 |
| 2 | 000114F | 48 | 44 | 23 | 0 | 41 | 84 | 3293850 | 3293981 | 4.00E-07 | 53 |
| 3 | 000135F | 31 | 74 | 50 | 1 | 21 | 94 | 994087 | 993869 | 5.00E-06 | 49 |
| 3 | 000152F | 46 | 68 | 35 | 2 | 21 | 86 | 2087390 | 2087187 | 8.00E-09 | 58 |
| 3 | 000152F | 47 | 68 | 34 | 2 | 21 | 86 | 2094071 | 2093868 | 6.00E-09 | 58 |
| 2 | 000171F | 48 | 44 | 23 | 0 | 41 | 84 | 107048 | 106917 | 6.00E-08 | 55 |
| 3 | 000219F | 67 | 24 | 8 | 0 | 71 | 94 | 100963 | 100892 | 2.00E-07 | 37 |
| 3 | 000219F | 65 | 26 | 9 | 0 | 49 | 74 | 101028 | 100951 | 2.00E-07 | 39 |
| 3 | 000219F | 89 | 35 | 4 | 0 | 21 | 55 | 101411 | 101307 | 4.00E-13 | 71 |
| 2 | 000219F | 41 | 37 | 22 | 0 | 4 | 40 | 136105 | 136215 | 3.00E-12 | 39 |
| 2 | 000219F | 53 | 47 | 20 | 1 | 46 | 90 | 136202 | 136342 | 3.00E-12 | 53 |
| 3 | 000219F | 85 | 33 | 5 | 0 | 19 | 51 | 136913 | 137011 | 5.00E-09 | 59 |
| 3 | 000219F | 74 | 27 | 7 | 0 | 48 | 74 | 144963 | 145043 | 8.00E-09 | 43 |
| 3 | 000219F | 67 | 24 | 8 | 0 | 71 | 94 | 145031 | 145102 | 8.00E-09 | 37 |
| 2 | 000219F | 54 | 50 | 23 | 0 | 38 | 87 | 147239 | 147090 | 2.00E-06 | 50 |
| 3 | 000219F | 32 | 74 | 50 | 0 | 21 | 94 | 221397 | 221618 | 7.00E-06 | 49 |
| 2 | 000219F | 54 | 59 | 27 | 0 | 2 | 60 | 246230 | 246054 | 3.00E-11 | 65 |
| 3 | 000219F | 63 | 24 | 9 | 0 | 71 | 94 | 267676 | 267605 | 2.00E-25 | 34 |
| 3 | 000219F | 85 | 55 | 8 | 0 | 20 | 74 | 267828 | 267664 | 2.00E-25 | 102 |
| 3 | 000219F | 34 | 74 | 49 | 0 | 21 | 94 | 331051 | 330830 | 3.00E-06 | 50 |
| 2 | 000219F | 52 | 33 | 16 | 0 | 2 | 34 | 484799 | 484897 | 1.00E-10 | 40 |
| 2 | 000219F | 46 | 48 | 26 | 0 | 38 | 85 | 484906 | 485049 | 1.00E-10 | 47 |
| 2 | 000219F | 46 | 48 | 26 | 0 | 38 | 85 | 507922 | 507779 | 1.00E-10 | 47 |
| 2 | 000219F | 52 | 33 | 16 | 0 | 2 | 34 | 508029 | 507931 | 1.00E-10 | 40 |
| 2 | 000220F | 55 | 58 | 26 | 0 | 2 | 59 | 427986 | 428159 | 3.00E-12 | 68 |
| 2 | 000220F | 53 | 58 | 27 | 0 | 2 | 59 | 466889 | 466716 | 9.00E-12 | 66 |
| 2 | 000220F | 53 | 58 | 27 | 0 | 2 | 59 | 508632 | 508805 | 2.00E-11 | 66 |
| 2 | 000220F | 54 | 54 | 25 | 0 | 2 | 55 | 511512 | 511351 | 3.00E-09 | 59 |
| 2 | 000227F | 52 | 33 | 16 | 0 | 2 | 34 | 237850 | 237948 | 6.00E-11 | 41 |
| 2 | 000227F | 46 | 48 | 26 | 0 | 38 | 85 | 237957 | 238100 | 6.00E-11 | 47 |
| 2 | 000227F | 46 | 48 | 26 | 0 | 38 | 85 | 292983 | 292840 | 1.00E-10 | 47 |
| 2 | 000227F | 52 | 33 | 16 | 0 | 2 | 34 | 293090 | 292992 | 1.00E-10 | 40 |
| 2 | 000227F | 46 | 48 | 26 | 0 | 38 | 85 | 362829 | 362686 | 8.00E-11 | 47 |
| 2 | 000227F | 52 | 33 | 16 | 0 | 2 | 34 | 362936 | 362838 | 8.00E-11 | 40 |
| 2 | 000227F | 52 | 33 | 16 | 0 | 2 | 34 | 409156 | 409254 | 2.00E-10 | 40 |
| 2 | 000227F | 46 | 48 | 26 | 0 | 38 | 85 | 409263 | 409406 | 2.00E-10 | 47 |
| 2 | 000227F | 47 | 53 | 28 | 0 | 38 | 90 | 497804 | 497962 | 5.00E-07 | 52 |
| 3 | 000232F | 75 | 24 | 6 | 0 | 71 | 94 | 48497 | 48426 | 5.00E-24 | 41 |
| 3 | 000232F | 77 | 56 | 10 | 1 | 19 | 74 | 48643 | 48485 | 5.00E-24 | 91 |
| 2 | 000232F | 48 | 48 | 25 | 0 | 38 | 85 | 111418 | 111275 | 3.00E-11 | 47 |
| 2 | 000232F | 55 | 33 | 15 | 0 | 2 | 34 | 111525 | 111427 | 3.00E-11 | 42 |
| 2 | 000232F | 48 | 44 | 23 | 0 | 41 | 84 | 166515 | 166646 | 7.00E-08 | 55 |

| | | | | | | | | | | | |
|---|---|---|---|---|---|---|---|---|---|---|---|
| 2 | 000232F | 48 | 44 | 23 | 0 | 41 | 84 | 183460 | 183329 | 7.00E-08 | 55 |
| 2 | 000239F | 53 | 40 | 19 | 0 | 41 | 80 | 116209 | 116328 | 3.00E-07 | 53 |
| 2 | 000239F | 51 | 41 | 20 | 0 | 41 | 81 | 135721 | 135843 | 1.00E-07 | 54 |
| 2 | 000239F | 36 | 70 | 45 | 0 | 15 | 84 | 179257 | 179048 | 5.00E-07 | 52 |
| 2 | 000239F | 48 | 44 | 23 | 0 | 41 | 84 | 201332 | 201463 | 8.00E-07 | 52 |
| 2 | 000239F | 39 | 70 | 43 | 0 | 15 | 84 | 214416 | 214625 | 6.00E-11 | 64 |
| 2 | 000239F | 52 | 27 | 13 | 0 | 8 | 34 | 330793 | 330873 | 6.00E-10 | 34 |
| 2 | 000239F | 45 | 49 | 27 | 0 | 37 | 85 | 330879 | 331025 | 6.00E-10 | 50 |
| 2 | 000239F | 48 | 44 | 23 | 0 | 41 | 84 | 384018 | 384149 | 2.00E-07 | 54 |
| 2 | 000245F | 43 | 44 | 25 | 0 | 41 | 84 | 38823 | 38954 | 3.00E-06 | 50 |
| 2 | 000250F | 52 | 33 | 16 | 0 | 2 | 34 | 21954 | 22052 | 1.00E-10 | 40 |
| 2 | 000250F | 46 | 48 | 26 | 0 | 38 | 85 | 22061 | 22204 | 1.00E-10 | 47 |
| 2 | 000250F | 52 | 33 | 16 | 0 | 2 | 34 | 80889 | 80987 | 1.00E-10 | 40 |
| 2 | 000250F | 46 | 48 | 26 | 0 | 38 | 85 | 80996 | 81139 | 1.00E-10 | 47 |
| 2 | 000250F | 46 | 48 | 26 | 0 | 38 | 85 | 167760 | 167617 | 1.00E-10 | 47 |
| 2 | 000250F | 52 | 33 | 16 | 0 | 2 | 34 | 167867 | 167769 | 1.00E-10 | 40 |
| 2 | 000250F | 46 | 48 | 26 | 0 | 38 | 85 | 188616 | 188473 | 1.00E-10 | 47 |
| 2 | 000250F | 52 | 33 | 16 | 0 | 2 | 34 | 188723 | 188625 | 1.00E-10 | 40 |
| 2 | 000250F | 52 | 33 | 16 | 0 | 2 | 34 | 217588 | 217686 | 1.00E-10 | 40 |
| 2 | 000250F | 46 | 48 | 26 | 0 | 38 | 85 | 217695 | 217838 | 1.00E-10 | 47 |
| 2 | 000251F | 52 | 33 | 16 | 0 | 2 | 34 | 28304 | 28402 | 9.00E-11 | 40 |
| 2 | 000251F | 46 | 48 | 26 | 0 | 38 | 85 | 28411 | 28554 | 9.00E-11 | 47 |
| 2 | 000251F | 66 | 89 | 30 | 0 | 2 | 90 | 76819 | 77085 | 1.00E-30 | 120 |
| 3 | 000251F | 83 | 54 | 9 | 0 | 21 | 74 | 77397 | 77558 | 7.00E-27 | 98 |
| 3 | 000251F | 75 | 24 | 6 | 0 | 71 | 94 | 77545 | 77616 | 7.00E-27 | 44 |
| 3 | 000257F | 100 | 76 | 0 | 0 | 19 | 94 | 18066 | 17839 | 8.00E-44 | 159 |
| 2 | 000257F | 83 | 90 | 15 | 0 | 1 | 90 | 19521 | 19252 | 2.00E-42 | 155 |
| 2 | 000258F | 46 | 48 | 26 | 0 | 38 | 85 | 71149 | 71006 | 1.00E-10 | 47 |
| 2 | 000258F | 52 | 33 | 16 | 0 | 2 | 34 | 71256 | 71158 | 1.00E-10 | 40 |
| 2 | 000258F | 52 | 33 | 16 | 0 | 2 | 34 | 143799 | 143897 | 1.00E-10 | 40 |
| 2 | 000258F | 46 | 48 | 26 | 0 | 38 | 85 | 143906 | 144049 | 1.00E-10 | 47 |
| 2 | 000258F | 46 | 48 | 26 | 0 | 38 | 85 | 168170 | 168027 | 9.00E-11 | 47 |
| 2 | 000258F | 52 | 33 | 16 | 0 | 2 | 34 | 168277 | 168179 | 9.00E-11 | 40 |
| 2 | 000258F | 46 | 52 | 28 | 0 | 38 | 89 | 222063 | 221908 | 1.00E-11 | 50 |
| 2 | 000258F | 52 | 33 | 16 | 0 | 2 | 34 | 222170 | 222072 | 1.00E-11 | 40 |
| 2 | 000264F_001 | 54 | 57 | 26 | 0 | 3 | 59 | 28478 | 28308 | 2.00E-11 | 66 |
| 2 | 000264F_005 | 52 | 58 | 28 | 0 | 2 | 59 | 5985 | 6158 | 8.00E-11 | 64 |
| 2 | 000264F_005 | 52 | 58 | 28 | 0 | 2 | 59 | 8863 | 8690 | 7.00E-11 | 64 |
| 2 | 000264F_005 | 55 | 58 | 26 | 0 | 2 | 59 | 25285 | 25112 | 8.00E-13 | 69 |
| 2 | 000264F_005 | 53 | 58 | 27 | 0 | 2 | 59 | 41788 | 41615 | 1.00E-11 | 66 |
| 2 | 000264F_005 | 53 | 58 | 27 | 0 | 2 | 59 | 70310 | 70483 | 7.00E-12 | 67 |
| 2 | 000264F_005 | 54 | 54 | 25 | 0 | 2 | 55 | 73186 | 73025 | 9.00E-10 | 61 |
| 2 | 000264F_005 | 53 | 58 | 27 | 0 | 2 | 59 | 97560 | 97733 | 1.00E-11 | 66 |
| 2 | 000264F_005 | 53 | 58 | 27 | 0 | 2 | 59 | 100451 | 100278 | 1.00E-11 | 66 |
| 2 | 000264F_005 | 53 | 58 | 27 | 0 | 2 | 59 | 136272 | 136099 | 3.00E-12 | 68 |
| 2 | 000264F_005 | 53 | 58 | 27 | 0 | 2 | 59 | 169011 | 169184 | 1.00E-11 | 66 |
| 2 | 000264F_005 | 52 | 58 | 28 | 0 | 2 | 59 | 171893 | 171720 | 8.00E-11 | 64 |
| 2 | 000264F_005 | 50 | 44 | 22 | 0 | 2 | 45 | 189479 | 189610 | 1.00E-06 | 51 |
| 2 | 000264F_007 | 52 | 58 | 28 | 0 | 2 | 59 | 4288 | 4461 | 1.00E-10 | 63 |

| | | | | | | | | | | | |
|---|---|---|---|---|---|---|---|---|---|---|---|
| 2 | 000264F_007 | 52 | 58 | 28 | 0 | 2 | 59 | 20033 | 20206 | 1.00E-10 | 63 |
| 2 | 000264F_007 | 53 | 58 | 27 | 0 | 2 | 59 | 22924 | 22751 | 1.00E-11 | 66 |
| 2 | 000276F | 36 | 70 | 45 | 0 | 15 | 84 | 87607 | 87816 | 3.00E-08 | 56 |
| 2 | 000276F | 39 | 69 | 42 | 0 | 16 | 84 | 151735 | 151529 | 1.00E-07 | 54 |
| 2 | 000295F | 48 | 44 | 23 | 0 | 41 | 84 | 19846 | 19977 | 1.00E-07 | 54 |
| 2 | 000295F | 48 | 44 | 23 | 0 | 41 | 84 | 70504 | 70635 | 2.00E-07 | 53 |
| 2 | 000295F | 45 | 44 | 24 | 0 | 41 | 84 | 91040 | 90909 | 8.00E-07 | 52 |
| 2 | 000295F | 48 | 44 | 23 | 0 | 41 | 84 | 116480 | 116611 | 2.00E-07 | 53 |
| 2 | 000295F | 48 | 44 | 23 | 0 | 41 | 84 | 153048 | 152917 | 1.00E-07 | 54 |
| 2 | 000309F | 45 | 44 | 24 | 0 | 41 | 84 | 92564 | 92695 | 5.00E-07 | 52 |
| 2 | 000315F | 48 | 44 | 23 | 0 | 41 | 84 | 36308 | 36177 | 4.00E-07 | 52 |
| 2 | 000315F | 48 | 44 | 23 | 0 | 41 | 84 | 56092 | 56223 | 2.00E-06 | 50 |
| 2 | 000332F | 50 | 44 | 22 | 0 | 41 | 84 | 60499 | 60630 | 2.00E-08 | 56 |
| 3 | 000344F | 31 | 74 | 51 | 0 | 21 | 94 | 34098 | 33877 | 3.00E-06 | 50 |
| 2 | 000348F | 52 | 33 | 16 | 0 | 2 | 34 | 19721 | 19819 | 7.00E-11 | 41 |
| 2 | 000348F | 46 | 48 | 26 | 0 | 38 | 85 | 19828 | 19971 | 7.00E-11 | 47 |
| 2 | 000348F | 46 | 48 | 26 | 0 | 38 | 85 | 40978 | 40835 | 1.00E-10 | 47 |
| 2 | 000348F | 52 | 33 | 16 | 0 | 2 | 34 | 41085 | 40987 | 1.00E-10 | 40 |
| 2 | 000353F | 53 | 58 | 27 | 0 | 2 | 59 | 15730 | 15903 | 5.00E-12 | 67 |
| 3 | 000357F | 34 | 74 | 49 | 0 | 21 | 94 | 47879 | 47658 | 6.00E-06 | 49 |
| 2 | 000357F | 99 | 90 | 1 | 0 | 1 | 90 | 54501 | 54770 | 7.00E-56 | 194 |
| 3 | 000357F | 100 | 76 | 0 | 0 | 19 | 94 | 55422 | 55649 | 2.00E-44 | 160 |
| 2 | 000357F | 98 | 90 | 2 | 0 | 1 | 90 | 69097 | 69366 | 5.00E-57 | 197 |
| 3 | 000357F | 100 | 76 | 0 | 0 | 19 | 94 | 70018 | 70245 | 2.00E-44 | 160 |
| 2 | 000357F | 74 | 90 | 23 | 0 | 1 | 90 | 80536 | 80805 | 3.00E-35 | 134 |
| 3 | 000357F | 100 | 76 | 0 | 0 | 19 | 94 | 81469 | 81696 | 9.00E-44 | 159 |
| 2 | 000358F | 38 | 84 | 52 | 0 | 2 | 85 | 32443 | 32694 | 9.00E-11 | 63 |
| 2 | 000366F | 52 | 58 | 28 | 0 | 2 | 59 | 9616 | 9789 | 8.00E-11 | 64 |
| 2 | 000366F | 53 | 58 | 27 | 0 | 2 | 59 | 12500 | 12327 | 3.00E-12 | 68 |
| 2 | 000366F | 52 | 58 | 28 | 0 | 2 | 59 | 28579 | 28406 | 1.00E-10 | 63 |
| 2 | 000366F | 52 | 56 | 27 | 0 | 4 | 59 | 44455 | 44288 | 1.00E-10 | 63 |
| 2 | 000366F | 53 | 58 | 27 | 0 | 2 | 59 | 77094 | 77267 | 9.00E-12 | 66 |
| 2 | 000369F | 53 | 58 | 27 | 0 | 2 | 59 | 2455 | 2282 | 2.00E-11 | 66 |
| 2 | 000369F | 53 | 58 | 27 | 0 | 2 | 59 | 41486 | 41313 | 1.00E-11 | 66 |
| 2 | 000369F | 53 | 58 | 27 | 0 | 2 | 59 | 58158 | 58331 | 1.00E-11 | 66 |
| 2 | 000369F | 53 | 58 | 27 | 0 | 2 | 59 | 61040 | 60867 | 1.00E-11 | 66 |
| 2 | 000369F | 53 | 58 | 27 | 0 | 2 | 59 | 77661 | 77834 | 1.00E-11 | 66 |
| 2 | 000369F | 53 | 58 | 27 | 0 | 2 | 59 | 80544 | 80371 | 2.00E-11 | 66 |
| 2 | 000371F | 45 | 47 | 26 | 0 | 41 | 87 | 31915 | 31775 | 1.00E-06 | 51 |
| 2 | 000375F | 48 | 44 | 23 | 0 | 41 | 84 | 73626 | 73495 | 3.00E-07 | 53 |
| 3 | 000381F | 75 | 24 | 6 | 0 | 71 | 94 | 64203 | 64132 | 7.00E-24 | 41 |
| 3 | 000381F | 77 | 56 | 10 | 1 | 19 | 74 | 64349 | 64191 | 7.00E-24 | 90 |
| 2 | 000410F | 48 | 44 | 23 | 0 | 41 | 84 | 67033 | 66902 | 2.00E-06 | 50 |
| 2 | 000413F | 46 | 48 | 26 | 0 | 38 | 85 | 19766 | 19623 | 1.00E-10 | 47 |
| 2 | 000413F | 52 | 33 | 16 | 0 | 2 | 34 | 19873 | 19775 | 1.00E-10 | 40 |
| 2 | 000413F | 52 | 33 | 16 | 0 | 2 | 34 | 70704 | 70802 | 9.00E-11 | 40 |
| 2 | 000413F | 46 | 48 | 26 | 0 | 38 | 85 | 70811 | 70954 | 9.00E-11 | 47 |
| 2 | 000421F | 52 | 27 | 13 | 0 | 8 | 34 | 40362 | 40442 | 8.00E-12 | 34 |
| 2 | 000421F | 47 | 53 | 28 | 0 | 38 | 90 | 40451 | 40609 | 8.00E-12 | 56 |

| | | | | | | | | | | | |
|---|---|---|---|---|---|---|---|---|---|---|---|
| 2 | 000421F | 48 | 44 | 23 | 0 | 41 | 84 | 79625 | 79756 | 2.00E-07 | 54 |
| 2 | 000447F | 46 | 48 | 26 | 0 | 38 | 85 | 32873 | 32730 | 1.00E-10 | 46 |
| 2 | 000447F | 52 | 33 | 16 | 0 | 2 | 34 | 32980 | 32882 | 1.00E-10 | 40 |
| 2 | 000454F_001 | 48 | 44 | 23 | 0 | 41 | 84 | 7404 | 7273 | 2.00E-07 | 54 |
| 2 | 000454F_001 | 45 | 44 | 24 | 0 | 41 | 84 | 30019 | 29888 | 3.00E-06 | 50 |
| 2 | 000485F_001 | 94 | 90 | 5 | 0 | 1 | 90 | 9214 | 9483 | 5.00E-52 | 182 |
| 3 | 000485F_001 | 100 | 76 | 0 | 0 | 19 | 94 | 10134 | 10361 | 3.00E-44 | 160 |
| 2 | 000485F_001 | 98 | 90 | 2 | 0 | 1 | 90 | 29737 | 30006 | 9.00E-57 | 196 |
| 3 | 000485F_001 | 100 | 76 | 0 | 0 | 19 | 94 | 30657 | 30884 | 3.00E-44 | 160 |
| 2 | 000485F_001 | 78 | 90 | 20 | 0 | 1 | 90 | 46119 | 46388 | 1.00E-39 | 147 |
| 3 | 000485F_001 | 100 | 76 | 0 | 0 | 19 | 94 | 46998 | 47225 | 8.00E-44 | 159 |
| 2 | 000501F | 53 | 58 | 27 | 0 | 2 | 59 | 301 | 474 | 3.00E-12 | 67 |
| 2 | 000501F | 53 | 58 | 27 | 0 | 2 | 59 | 3183 | 3010 | 1.00E-11 | 66 |
| 2 | 000501F | 53 | 58 | 27 | 0 | 2 | 59 | 30031 | 30204 | 9.00E-12 | 66 |
| 2 | 000542F | 52 | 56 | 27 | 0 | 4 | 59 | 7230 | 7063 | 1.00E-10 | 63 |
| 2 | 000577F | 48 | 44 | 23 | 0 | 41 | 84 | 35282 | 35413 | 3.00E-08 | 56 |
| 2 | 000826F | 48 | 44 | 23 | 0 | 41 | 84 | 11352 | 11221 | 2.00E-08 | 56 |
| 2 | 000868F | 66 | 87 | 29 | 1 | 1 | 87 | 4152 | 4409 | 7.00E-29 | 115 |
| 3 | 000868F | 96 | 56 | 2 | 0 | 19 | 74 | 5021 | 5188 | 9.00E-36 | 115 |
| 3 | 000868F | 100 | 24 | 0 | 0 | 71 | 94 | 5176 | 5247 | 9.00E-36 | 56 |
| 2 | 000868F | 98 | 90 | 2 | 0 | 1 | 90 | 15490 | 15759 | 2.00E-55 | 192 |
| 3 | 000868F | 100 | 76 | 0 | 0 | 19 | 94 | 16410 | 16637 | 3.00E-44 | 160 |
| 2 | 000868F | 98 | 90 | 1 | 1 | 1 | 90 | 26421 | 26687 | 2.00E-54 | 189 |
| 3 | 000868F | 100 | 76 | 0 | 0 | 19 | 94 | 27337 | 27564 | 2.00E-44 | 160 |
| 2 | 000952F_002 | 52 | 33 | 16 | 0 | 2 | 34 | 13410 | 13508 | 1.00E-10 | 40 |
| 2 | 000952F_002 | 46 | 48 | 26 | 0 | 38 | 85 | 13517 | 13660 | 1.00E-10 | 47 |

**Table S6.** Analysis of runs of homozygosity (ROH) for Alala (*Corvus hawaiiensis*, Coha) and Anna's hummingbird (*Calypte anna*, Caan) [29] genome contigs of minimum length 500 kb ($n$ = 209 and $n$ = 283, respectively). The ROH segment length is the cumulative length of uninterrupted 100 kb sliding windows (SW) that were completely autozygous or fell within one of four heterozygosity thresholds. The autozygous fraction of the genome (fROH) is calculated as the total number of sliding windows in ROHs (# SW-ROH) divided by the total number of sliding windows ($n$ = 10,338 Coha; $n$ = 9373 Caan) across all genome contigs.

| | **Number ROHs per length category** | | | | | | | | | |
|---|---|---|---|---|---|---|---|---|---|---|
| ROH Segment Length (kb) | 0 hets / 100kb | | ≤1 het / 100kb | | ≤1 het / 50kb | | ≤1 het / 25kb | | ≤1 het / 10 kb | |
| | Coha | Caan | Coha | Caan | Coha | Caan | Coha | Caan | Coha | Caan |
| 100 | 464 | 80 | 840 | 54 | 742 | 50 | 280 | 43 | 159 | 72 |
| 200 | 42 | 37 | 223 | 24 | 334 | 19 | 165 | 15 | 57 | 14 |
| 300 | 6 | 12 | 72 | 14 | 186 | 11 | 113 | 9 | 27 | 7 |
| 400 | 2 | 16 | 25 | 15 | 89 | 11 | 100 | 10 | 22 | 9 |
| 500 | 0 | 18 | 7 | 13 | 56 | 9 | 68 | 11 | 9 | 9 |
| 600 | 0 | 6 | 2 | 8 | 21 | 10 | 55 | 8 | 9 | 11 |
| 700 | 0 | 1 | 1 | 4 | 14 | 6 | 52 | 5 | 14 | 4 |
| 800 | 0 | 1 | 0 | 2 | 5 | 6 | 31 | 4 | 13 | 7 |
| 900 | 0 | 0 | 0 | 2 | 4 | 2 | 30 | 4 | 8 | 3 |
| 1000 | 0 | 1 | 0 | 4 | 3 | 4 | 24 | 3 | 9 | 4 |
| 1100 | 0 | 1 | 0 | 2 | 0 | 2 | 21 | 2 | 6 | 2 |
| 1200 | 0 | 0 | 0 | 0 | 1 | 0 | 18 | 1 | 9 | 1 |
| 1300 | 0 | 0 | 0 | 0 | 0 | 2 | 9 | 1 | 4 | 2 |
| 1400 | 0 | 0 | 0 | 0 | 0 | 0 | 10 | 1 | 4 | 1 |
| 1500 | 0 | 0 | 0 | 0 | 0 | 0 | 9 | 1 | 3 | 0 |
| 1600 | 0 | 0 | 0 | 1 | 0 | 2 | 6 | 2 | 4 | 2 |
| 1700 | 0 | 0 | 0 | 1 | 0 | 1 | 7 | 2 | 5 | 2 |
| 1800 | 0 | 0 | 0 | 0 | 0 | 0 | 3 | 0 | 6 | 0 |
| 1900 | 0 | 0 | 0 | 2 | 0 | 1 | 6 | 0 | 5 | 1 |
| 2000 | 0 | 0 | 0 | 0 | 0 | 0 | 2 | 0 | 2 | 0 |
| 2100 | 0 | 0 | 0 | 0 | 0 | 0 | 1 | 0 | 3 | 0 |
| 2200 | 0 | 0 | 0 | 0 | 0 | 0 | 3 | 1 | 3 | 0 |
| 2300 | 0 | 0 | 0 | 0 | 0 | 1 | 2 | 1 | 2 | 1 |
| 2400 | 0 | 0 | 0 | 0 | 0 | 0 | 1 | 0 | 2 | 1 |
| 2500 | 0 | 0 | 0 | 0 | 0 | 0 | 1 | 0 | 3 | 0 |
| 2600 | 0 | 0 | 0 | 0 | 0 | 0 | 0 | 1 | 0 | 0 |
| 2700 | 0 | 0 | 0 | 0 | 0 | 0 | 1 | 0 | 1 | 0 |
| 2800 | 0 | 0 | 0 | 0 | 0 | 0 | 2 | 0 | 1 | 0 |
| 2900 | 0 | 0 | 0 | 0 | 0 | 0 | 0 | 0 | 1 | 0 |
| 3000 | 0 | 0 | 0 | 0 | 0 | 0 | 1 | 0 | 4 | 0 |
| 3100 | 0 | 0 | 0 | 0 | 0 | 0 | 0 | 0 | 1 | 0 |
| 3200 | 0 | 0 | 0 | 0 | 0 | 0 | 0 | 0 | 1 | 0 |
| 3300 | 0 | 0 | 0 | 0 | 0 | 0 | 1 | 0 | 2 | 0 |
| 3400 | 0 | 0 | 0 | 0 | 0 | 0 | 0 | 0 | 1 | 0 |

| | | | | | | | | | | |
|---|---|---|---|---|---|---|---|---|---|---|
| 3500 | 0 | 0 | 0 | 0 | 0 | 0 | 0 | 0 | 0 | 0 |
| 3600 | 0 | 0 | 0 | 0 | 0 | 0 | 0 | 0 | 3 | 0 |
| 3700 | 0 | 0 | 0 | 0 | 0 | 0 | 0 | 0 | 0 | 1 |
| 3800 | 0 | 0 | 0 | 0 | 0 | 0 | 1 | 0 | 0 | 1 |
| 3900 | 0 | 0 | 0 | 0 | 0 | 0 | 0 | 0 | 2 | 0 |
| 4000 | 0 | 0 | 0 | 0 | 0 | 0 | 0 | 0 | 3 | 0 |
| 4100 | 0 | 0 | 0 | 0 | 0 | 0 | 0 | 0 | 1 | 0 |
| 4200 | 0 | 0 | 0 | 0 | 0 | 0 | 0 | 0 | 1 | 0 |
| 4300 | 0 | 0 | 0 | 0 | 0 | 0 | 0 | 0 | 3 | 0 |
| 4500 | 0 | 0 | 0 | 0 | 0 | 0 | 0 | 0 | 1 | 0 |
| 4600 | 0 | 0 | 0 | 0 | 0 | 0 | 0 | 0 | 1 | 0 |
| 4700 | 0 | 0 | 0 | 0 | 0 | 0 | 0 | 0 | 1 | 0 |
| 4800 | 0 | 0 | 0 | 0 | 0 | 0 | 0 | 0 | 3 | 0 |
| 4900 | 0 | 0 | 0 | 0 | 0 | 0 | 0 | 0 | 1 | 0 |
| 5100 | 0 | 0 | 0 | 0 | 0 | 0 | 0 | 0 | 1 | 0 |
| 5200 | 0 | 0 | 0 | 0 | 0 | 0 | 0 | 0 | 1 | 0 |
| 5400 | 0 | 0 | 0 | 0 | 0 | 0 | 0 | 0 | 4 | 0 |
| 5500 | 0 | 0 | 0 | 0 | 0 | 0 | 0 | 0 | 2 | 0 |
| 5800 | 0 | 0 | 0 | 0 | 0 | 0 | 0 | 0 | 1 | 0 |
| 6300 | 0 | 0 | 0 | 0 | 0 | 0 | 0 | 0 | 2 | 0 |
| 6400 | 0 | 0 | 0 | 0 | 0 | 0 | 0 | 0 | 2 | 0 |
| 6500 | 0 | 0 | 0 | 0 | 0 | 0 | 0 | 0 | 1 | 0 |
| 6600 | 0 | 0 | 0 | 0 | 0 | 0 | 0 | 0 | 1 | 0 |
| 7000 | 0 | 0 | 0 | 0 | 0 | 0 | 0 | 0 | 1 | 0 |
| 7500 | 0 | 0 | 0 | 0 | 0 | 0 | 0 | 0 | 1 | 0 |
| 7600 | 0 | 0 | 0 | 0 | 0 | 0 | 0 | 0 | 1 | 0 |
| 7700 | 0 | 0 | 0 | 0 | 0 | 0 | 0 | 0 | 1 | 0 |
| 8000 | 0 | 0 | 0 | 0 | 0 | 0 | 0 | 0 | 1 | 0 |
| 8500 | 0 | 0 | 0 | 0 | 0 | 0 | 0 | 0 | 1 | 0 |
| 8600 | 0 | 0 | 0 | 0 | 0 | 0 | 0 | 0 | 1 | 0 |
| 8900 | 0 | 0 | 0 | 0 | 0 | 0 | 0 | 0 | 1 | 0 |
| 9300 | 0 | 0 | 0 | 0 | 0 | 0 | 0 | 0 | 1 | 0 |
| 10,300 | 0 | 0 | 0 | 0 | 0 | 0 | 0 | 0 | 1 | 0 |
| 14,300 | 0 | 0 | 0 | 0 | 0 | 0 | 0 | 0 | 1 | 0 |
| 14,700 | 0 | 0 | 0 | 0 | 0 | 0 | 0 | 0 | 1 | 0 |
| 18,400 | 0 | 0 | 0 | 0 | 0 | 0 | 0 | 0 | 1 | 0 |
| 19,100 | 0 | 0 | 0 | 0 | 0 | 0 | 0 | 0 | 1 | 0 |
| 24,900 | 0 | 0 | 0 | 0 | 0 | 0 | 0 | 0 | 1 | 0 |
| # SW-ROH | 574 | 416 | 1656 | 512 | 2496 | 557 | 4769 | 589 | 5886 | 700 |
| fROH | 0.056 | 0.054 | 0.160 | 0.055 | 0.285 | 0.059 | 0.461 | 0.063 | 0.569 | 0.075 |

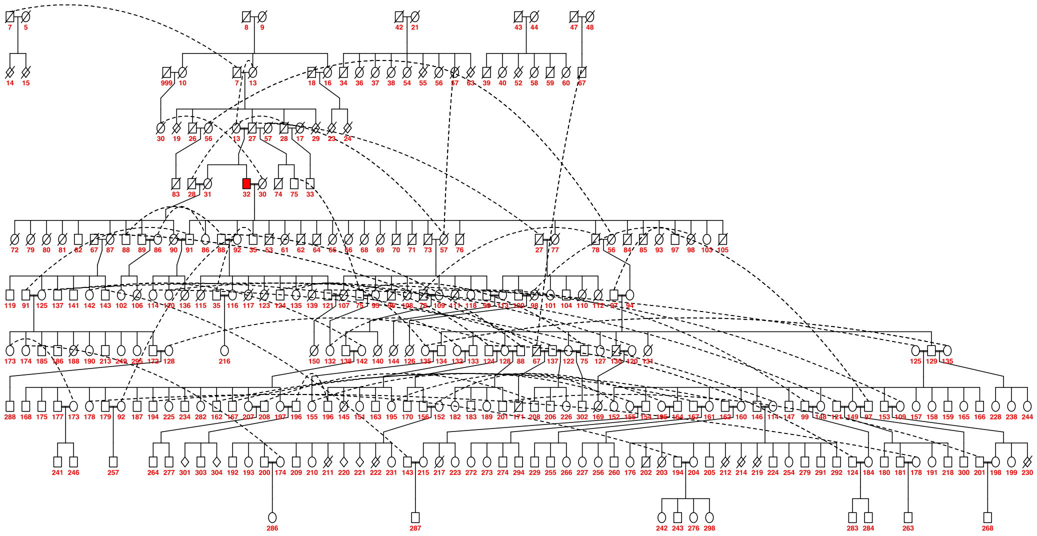

**Figure S1**. ʻAlalā pedigree. The sequenced individual, studbook 32 (named Hōʻike i ka pono) is shaded. Dashed lines indicate individuals that are represented in multiple positions in the pedigree (e.g., overlapping generations).

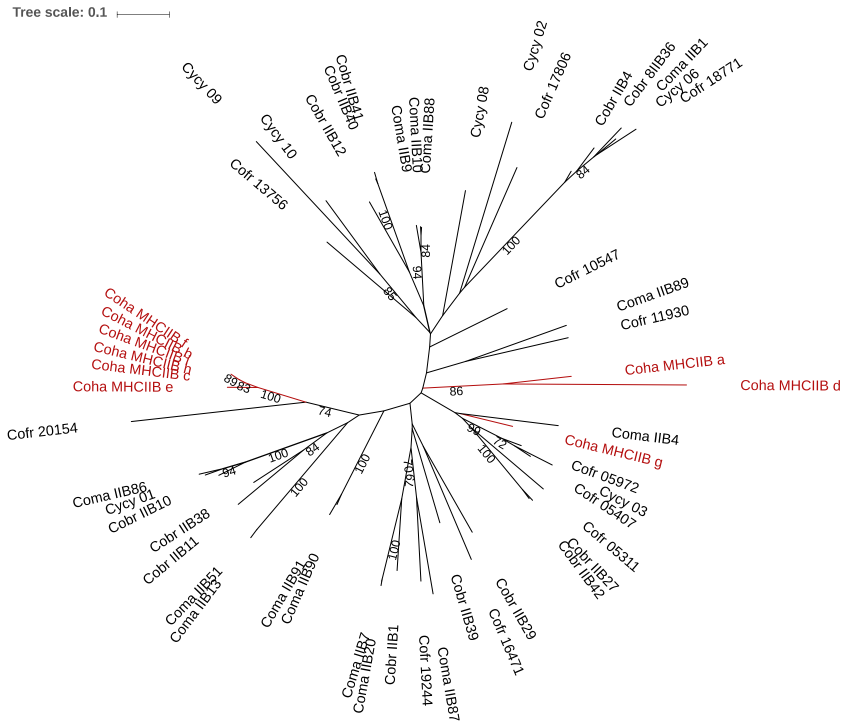


**Figure S2.** Unrooted maximum likelihood tree of corvid MHC class II B genes, exon 2. Functional genes predicted from the ʻAlalā assembly are highlighted in red (prefix Coha). Other species represented include *C. brachyrhynchos* (American crow, Cobr), *C. macrorhynchos* (jungle crow, Coma), *C. frugilegus* (Asian rook, Cofr) and *Cyanopica cyanus* (azure-winged magpie, Cycy). All genes were obtained from one individual per species [47]. Confidence values are given for nodes with bootstrap support >70%.
